# Fibro-Osseous Lesion of the Nose and Paranasal Sinus: A Retrospective Study With Literature Review

**DOI:** 10.7759/cureus.27229

**Published:** 2022-07-25

**Authors:** Nasir A Magboul, Mohammad S Al-Ahmari, Mohammed A Alzahrani, Shahad S Dlboh

**Affiliations:** 1 Department of Otolaryngology - Head and Neck Surgery, Armed Forces Hospital, Jazan, SAU; 2 Department of Otolaryngology - Head and Neck Surgery, Aseer Central Hospital, Abha, SAU

**Keywords:** osteoma, fibro-osseous lesion, endoscopic surgery, paranasal, fibrous dysplasia, ossifying fibroma

## Abstract

Background

Fibro-osseous (FO) lesions are slow-growing benign lesions in the paranasal sinuses. They include osteomas, fibrous dysplasia (FD), and ossifying fibro­ma (OF). Fibro-osseous (FO) lesions are frequently asymptomatic, and they are incidentally found on imaging. They are characterized by different histological, radiological, and clinical variants. Depending on symptoms, size, location, and extension, the treatment strategy varies significantly for these lesions.

Objective

We aim to compare the age, onset, gender, clinical presentation, postoperative improvement, and complications of a fibro-osseous lesion in the paranasal sinuses.

Methods

A retrospective analysis was done targeting patients diagnosed with benign fibro-osseous (FO) lesions, and the incidence among 403 patients who underwent functional endoscopic sinus surgery (FESS) at Aseer Central Hospital, Kingdom of Saudi Arabia, was reviewed from January 2013 to January 2022.

Results

A total of seven patients were found; five patients were diagnosed with osteoma, and two were diagnosed with fibrous dysplasia. There were no ossifying fibroma cases. The patients’ mean age was 25.5 ± 12.9 years old. Four (57.1%) patients were males, and three (42.9%) were females, with a male/female ratio of 1.25:1. The most common locations were the frontal sinus and ethmoid sinus, and the two cases of fibrous dysplasia involved almost all facial bones. The endonasal endoscopic approach was chosen to treat all seven patients.

Conclusions

There are differences in the onset age, location, and complications postoperatively among osteoma and fibrous dysplasia patients. Osteoma most commonly occurs in the frontal sinus, while fibrous dysplasia involved all facial bones in our study. Endoscopic surgery is currently the primary strategy for treatment.

## Introduction

Fibro-osseous (FO) lesions are slow-growing benign lesions in the paranasal sinuses [1]. They are divided into osteomas, fibrous dysplasia (FD), and ossifying fibro­ma (OF) [1]. Among them, osteoma lesions are considered the most common benign tumors of the paranasal sinuses [2]. Fibro-osseous lesions are frequently asymptomatic, where they are incidentally found on imaging taken for another reason. However, they are benign lesions and, if extended, may cause ocular complications such as diplopia and proptosis [1]. They are characterized by different histological and radiological variants [2]. Because of the ossified and fibrous contents of fibro-osseous lesions, the diagnosis is sometimes difficult to obtain and depends only on imaging. In most cases, a thorough examination of histological, clinical, and radiological evidence is required for a correct diagnosis [2].

## Materials and methods

Methodology

A record-based retrospective study targeted benign fibro-osseous (FO) lesions, and the incidence among 403 patients at Aseer Central Hospital was reviewed from January 2013 to January 2022. Data were collected through phone calls and reviewing the electronic medical files for demographics, clinical presentation, postoperative improvement, postoperative complications, imaging, surgical approach, and follow-up. Data were extracted using pre-structured data extraction sheet to minimize interobserver bias and data extraction error. CT of the paranasal sinuses was obtained pre- and postoperatively. All the cases of osteoma were successfully removed without significant operative complications using an endoscopic approach as a modality of choice.

Data analysis

After data were extracted, it was revised, coded, and fed to the Statistical Package for Social Sciences (SPSS) version 22.0 (IBM Corp., Armonk, NY, USA). Descriptive analysis was done for all variables of the study cases with row data presentation, including the patients’ age, gender, clinical presentation, postoperative improvement, and complications besides follow-up data.

## Results

Among our institute’s 403 endoscopic sinus surgeries, we found seven cases of fibro-osseous lesions with an incidence of 1.73%. Among those cases, the final histological diagnosis showed five cases of osteoma and two cases of fibrous dysplasia, and there are no ossifying fibroma cases. The patients’ ages ranged from 10 to 50 years, with a mean age of 25.5 ± 12.9 years old. Four (57.1%) patients were males, and three (42.9%) were females, with a male/female ratio of 1.25:1 (Table 1).

**Table 1:** Bio-clinical features of the study patients

Patients	Sex	Age	Symptoms	Most common symptoms	Size of the tumors	Most affected sinus	Histological diagnosis	Complications after surgery
1	Male	50	Nasal obstruction, sneezing, headache, snoring	Headache	1.5 cm	Left frontal	Osteoma	None
2	Female	30	Nasal obstruction	Nasal obstruction	1.5 × 1.5 × 0.8 cm	Right frontal	Osteoma	None
3	Male	16	Nasal obstruction, headache, periorbital swelling, visual field defects	Headache	2 cm	Left frontal	Osteoma	None
4	Female	20	Nasal obstruction, headache	Nasal obstruction, headache	0.8 × 0.7 × 0.6 cm	Right ethmoid	Osteoma	Hyposmia
5	Female	29	Nasal obstruction, headache	Nasal obstruction, headache	1 × 0.6 × 0.5 cm	Left ethmoid	Osteoma	None
6	Male	10	Nasal obstruction, headache, seizure	Nasal obstruction, headache	Pieces largest 2.5 × 1.8 × 1.4 cm	Skull and facial bones	Fibrous dysplasia	None
7	Male	24	Nasal obstruction	Nasal obstruction	Diffusely involved the face	Facial bones	Fibrous dysplasia	None

Nasal obstruction was reported among all cases (100%). Headache was among five (71.4%) patients, while other clinical symptoms, including sneezing, snoring, periorbital swelling, proptosis, visual defects, and seizures, were detected in one case for each. The most affected sinus was the frontal sinus (three out of seven), especially the left frontal sinus.

**Figure 1:**
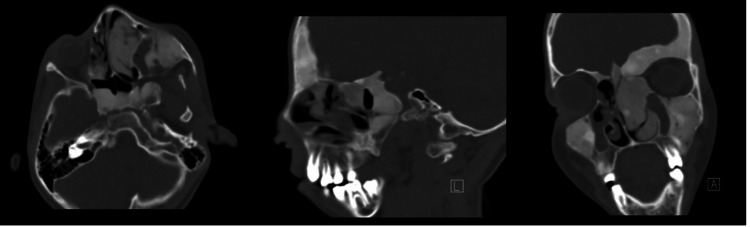
Fibrous dysplasia Plain CT scan of the paranasal sinus showing hyperostosis of the skull and facial bone with areas of sclerosis and ground-glass appearance. Biopsy was taken only from the inferior turbinate, confirming the diagnosis.

**Figure 2:**
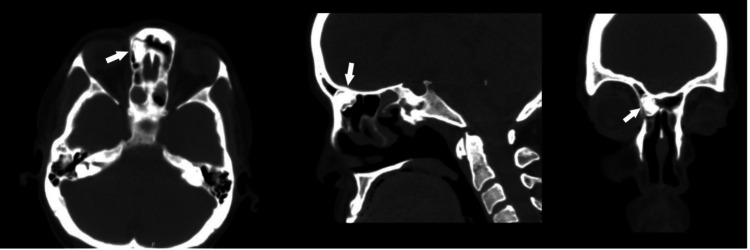
Preoperative CT scan Right frontal sinus osteoma (arrow) in axial, sagittal, and coronal views.

**Figure 3:**
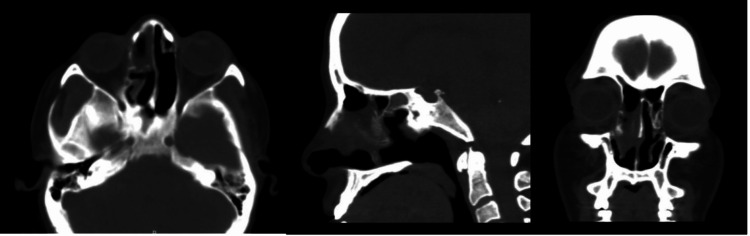
Postoperative CT scan Right frontal sinus osteoma in postoperative view.

**Figure 4:**
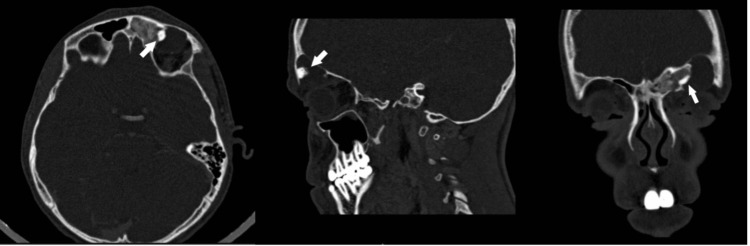
Preoperative CT scan Left frontal sinus osteoma (arrow) in axial, sagittal, and coronal views.

**Figure 5:**
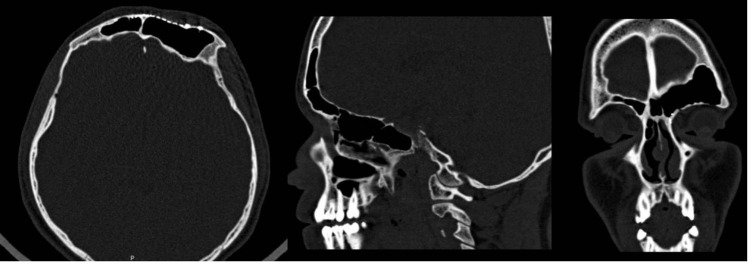
Postoperative CT scan Left frontal sinus osteoma in postoperative view.

The tumor size ranged from 0.8 cm to 2 cm. Considering the CT finding, two cases of fibrous dysplasia showed a ground-glass appearance, and the other five cases of osteoma showed a homogenous hyperdense mass with defined margins. Pathology showed fibrous dysplasia in two cases and osteoma in the other five cases. Functional endoscopic sinus surgery (FESS) was the procedure of choice among the whole cases of osteoma. None of the lesions has intracranial or intra-orbital extension. Regarding the fibrous dysplasia cases, only biopsy and debulking of the lesion were done (Figures 1-5).

As for postsurgical complications, the vast majority (85.7%; six cases) had no complications, while only one case experienced hyposmia. The patients are seen in postoperative visits with no recurrence of symptoms or signs. The most reported clinical symptoms among males and females after nasal obstruction were headaches (75% and 66.7%, respectively). The only postsurgical complication (hyposmia) was reported for a female patient aged 20 years.

## Discussion

The nose and paranasal sinuses can be affected by a wide range of disorders including inflammatory, infectious, and neoplastic disorders, among which a class of benign bony neoplasm known as fibro-osseous lesions. Fibrous dysplasia, ossifying fibroma, and osteoma are three different lesions that range from the least to the most bony content [3,4]. These lesions share standard features and appearance, with variant clinical implications [5]. Osteoma is the most common tumor of the paranasal sinuses and the most common fibro-osseous lesion. Fibrous dysplasia and ossifying fibroma (OF) are less frequently affecting the nose and paranasal sinus. The reported incidence of osteoma ranged from 0.014% to 0.43% [6-10]. Histologically, osteoma formed from the proliferation of compact, lamellar cortical bone. Therefore, radiographical features appear as well-circumscribed dense masses attached to the surrounding bone by either a broad narrow-based pedicle [11,12]. Histologically, osteoma can be divided into three types. First is an ivory type that contains dense cortical bone. The second type is a spongy form formed of cancellous bone, and the third type is a mix of the first two types [13-15]. The current study focused on fibro-osseous lesions of the nose and paranasal sinuses through the description of study cases and literature review.

In a case series study concluded by Flora et al. of 600 patients with nasal polyposis, 20 cases of osteoma were diagnosed, with an incidence of 3.3%, and the mean age was 45.7 ± 16.7 years, which is higher than our study patients’ ages [1]. Males were affected more than females, with a ratio of 2.3:1. In 12 patients representing 70% of cases of osteoma, the lesion was located in the frontal sinus. Seven (35%) were found on the lateral part of the ethmoid roof and one (5%) in the frontoethmoid area. The average tumor size was 11.04 ± 8.16 mm. The most identified feature was chronic mucosal inflammation, including bone modification, which finally causes osteomas. Mehta et al. reported two aggressive fibro-osseous lesions arising from paranasal sinuses and the anterior skull base in childhood with good surgical management [4]. The authors concluded that fibro-osseous lesions’ clinical behavior and radiological features were diverse. Aggressive lesions need a radical surgical intervention to confirm the complete excision, despite increased associated morbidity. Disease recurrence is the highest hazard with incomplete excision of aggressive lesions with higher morbidity or mortality.

On the other hand, a slowly progressive lesion often does not require extensive surgical excision. Alturaiki et al. review all cases of fibro-osseous lesions in the nose and paranasal sinuses [16]. They found that osteomas were found at the frontal sinuses in 40% of cases (four patients), sphenoid sinus in 20% (two patients); other two cases were found in the frontoethmoidal area, and one case involved the ethmoid sinus (10%). Males were affected four times more than females, higher than the current study findings. All their cases of osteoma underwent an endoscopic approach, and no reported intracranial or intra-orbital complications, with no evidence of recurrence of their osteomas in the follow-up. There was no clinical evidence of residual or recurrence in the osteoma cases. We lost follow-up after 36 months.

Sun et al. reported on a 46-year-old female patient with ossifying fibroma [17]. CT scan showed dilation of the right frontal sinus and dense masses in the cavity. A combined approach was chosen, frontal sinus fenestration and an endoscopic approach, to address the frontal recess. The postoperative pathological diagnosis was confirmed as an ossifying fibroma. Upon follow-up of five years, there was no clinical evidence of recurrence.

In our study, only biopsy and debulking of the lesion were done in two cases of fibrous dysplasia. Follow-up was lost after the third postoperative visit for the two patients. Extra sinus complications are classified as orbital and intracranial problems. Osteomas may slowly progress into the orbital vault, displacing the orbital contents. This entity can be clinically presented by diplopia, epiphora, facial distortion, and even blindness [18-22]. The removal of the osteoma usually restores normal vision. In rare instances of extreme expansion, the orbital vault requires reconstruction.

Limitation

This was a retrospective record-based study with all the limitations accompanying its design. Moreover, the study was also limited to only one hospital, with only the patients whose information was found in the new system used by the hospital. Follow-up was lost in two cases of fibrous dysplasia and was limited to three postoperative visits.

## Conclusions

In conclusion, besides the literature review, the current study findings conclude a good prognosis for these cases. Also, these cases are more frequent among males with different radiological features. The endoscopic approach is a safe and effective treatment modality with minimal complication and low recurrent complete resection of the tumor. Recurrence mainly occurs due to incomplete surgery.
